# Environmental variables driving horseshoe crab spawning behavior in a microtidal lagoon in Florida

**DOI:** 10.1371/journal.pone.0302433

**Published:** 2024-06-12

**Authors:** Berlynna Heres, Holly Abeels, Colin Shea, Claire E. Crowley-McIntyre

**Affiliations:** 1 Fish and Wildlife Research Institute, Florida Fish and Wildlife Conservation Commission, St. Petersburg, Florida, United States of America; 2 University of Florida Institute of Food and Agricultural Sciences Brevard County, Florida SeaGrant, Cocoa, Florida, United States of America; Universidade de Aveiro, PORTUGAL

## Abstract

The timing of American horseshoe crab (*Limulus polyphemus*) spawning behavior along the coast of Florida (United States) is generally associated with the highest tides during the spring and fall lunar cycles. All Florida estuaries support horseshoe crab populations, but tidal characteristics vary markedly among locations, which may influence the timing of horseshoe crab spawning behavior. The Indian River Lagoon is a large microtidal estuary on Florida’s east coast. Given the microtidal nature of the lagoon, it is unclear which environmental factors affect horseshoe spawning. In 2019, volunteers of Florida Horseshoe Crab Watch conducted daily surveys at two sites in the northern Indian River Lagoon during peak spawning months (February–April). During each survey, volunteers counted all spawning horseshoe crabs and recorded environmental variables, including water temperature, air temperature, wind speed, wind direction, salinity, and tide height. We developed a suite of negative-binomial regression models to quantify relationships between the number of spawning horseshoe crabs and environmental factors. Modeling results indicated a positive relationship between onshore wind speed and number of spawning horseshoe crabs. Our study suggests that in the absence of tidal cues, onshore wind speed may be an important driver of horseshoe crab spawning activity in microtidal estuarine systems.

## Introduction

The American horseshoe crab (*Limulus polyphemus*) is found throughout most of the Atlantic Coast and Gulf of Mexico of the United States from Maine to Alabama and in the Yucatan Peninsula. Despite the species’ wide-ranging distribution, the timing of spawning is closely linked with similar endogenous natural cues throughout much of its range [1, 2]. Horseshoe crabs are known to synchronize spawning in response to the lunar and tidal cycles, temperature, and wind direction [2, 3]. Tidal cycles, although predictable, are not dependent solely on lunar and solar cycles. The topography of the ocean bottom and curvature of the shoreline also influence the size and period of the tides [4]. The highest tides of the spring, which generally coincide with full and new moons, pile water higher onto the shore, which in turn provides ideal nesting conditions for spawning horseshoe crabs [5–7]. Horseshoe crabs nest in pairs, or in larger spawning congregations, along sandy slightly sloped beaches at the highest point of the high-tide line, which provides ideal sand moisture for the growth and development of their eggs, ensuring that the eggs do not become desiccated or the soil anoxic [8].

The Indian River Lagoon, a 250-km-long complex of three subbasins, the Indian River, the Banana River, and Mosquito Lagoon, is the largest microtidal estuary in Florida, United States [9]. Microtidal coasts are defined as those for which the tidal range—the distance between mean highest high tide and mean lowest low tide—does not exceed 2 m [10]. Horseshoe crab spawning in the Indian River Lagoon is unpredictable and aperiodic with some correlation with wind and temperature [11, 12], rather than tidal correspondence seen through much of Florida [3]. In similar microtidal estuaries, such as St. Joseph Bay, Florida, spawning was found to be triggered by an increase in water level [13], water level was not associated with increased spawning in the Indian River Lagoon [11]. The microtidal nature of the Indian River Lagoon makes it difficult to predict spawning of horseshoe crabs in this region. Continued costal development and shoreline hardening in the region make establishing baseline population information even more difficult, as habitat loss and other physical factors are difficult to account for in addition to the microtidal properties of the lagoon. Horseshoe crabs serve an important role within the food web, especially for shorebirds, and shifting populations could have negative repercussions to the ecosystem [12, 14].

Florida Horseshoe Crab Watch, a collaborative effort of the Florida Fish and Wildlife Conservation Commission’s Fish and Wildlife Research Institute (FWC-FWRI), the University of Florida’s Institute of Food and Agricultural Science Nature Coast Biological Station, and the University of Florida’s Department of Biology, was established in 2015 and uses citizen scientists to survey and tag spawning horseshoe crabs [14, 15]. Florida Horseshoe Crab Watch requires volunteers to undergo training, and all data are thoroughly vetted at the end of every survey season; consequently, the program has produced data equivalent to that of professional researchers [15]. When establishing survey locations for this program, public reports, compiled by FWC-FWRI, as described in Heres et al. [15], were used to select the sites where horseshoe crabs were most likely to spawn. Anecdotal reports and historical research indicated a high density of horseshoe crabs at two northern Indian River Lagoon microtidal sites between February and April which were incorporated into the Florida Horseshoe Crab Watch program [12]. Although night spawning has been reported, the surveys were performed during the day for the safety of volunteers. The surveys were performed by starting at a designated point on the transect, and walking at a steady pace, counting all horseshoe crabs visible in a single pass to a designated stopping point. Environmental variables were collected immediately after the transect was surveyed, or collected concurrently if there were multiple volunteers on the survey [15].

Throughout the state, Florida Horseshoe Crab Watch surveys coincide with the maximum high tide, even in microtidal estuaries. During surveys, volunteers recorded environmental data to determine what factors best predicted spawning in horseshoe crab in the northern Indian River Lagoon [14, 15]. We hypothesized that when the tide was not the predominant force guiding spawning, wind and wind direction would be the strongest contributing factors.

## Methods

### Study location

**Fig 1:**
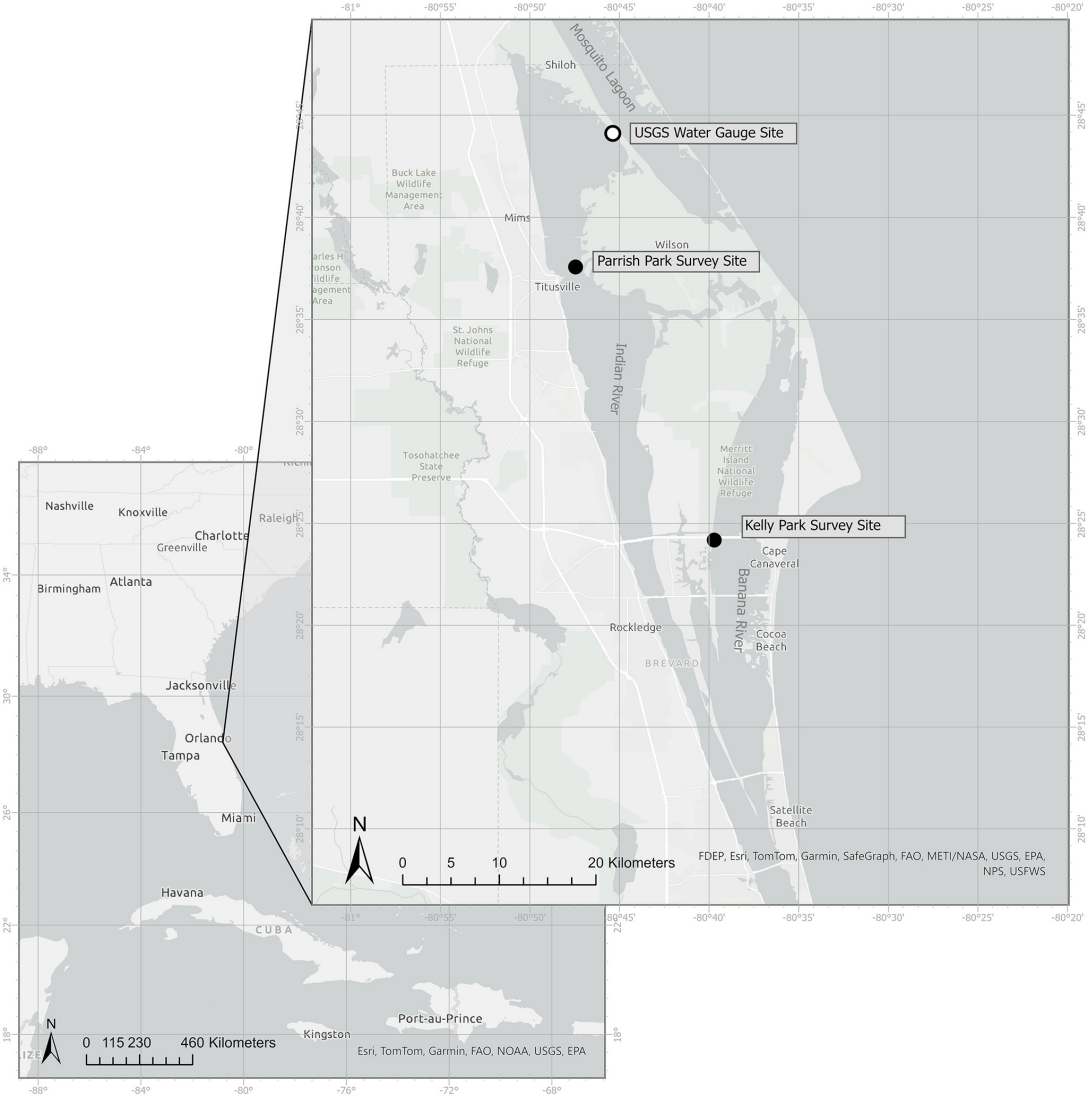
Location of survey sites. Location of survey sites in the northern Indian River Lagoon, Florida. Created with ESRI ArcPro software 3.1.2. Content is the intellectual property of ESRI and is used herein with permission. Copyright © 2024 Esri and its licensors. All rights reserved.

**Fig 2:**
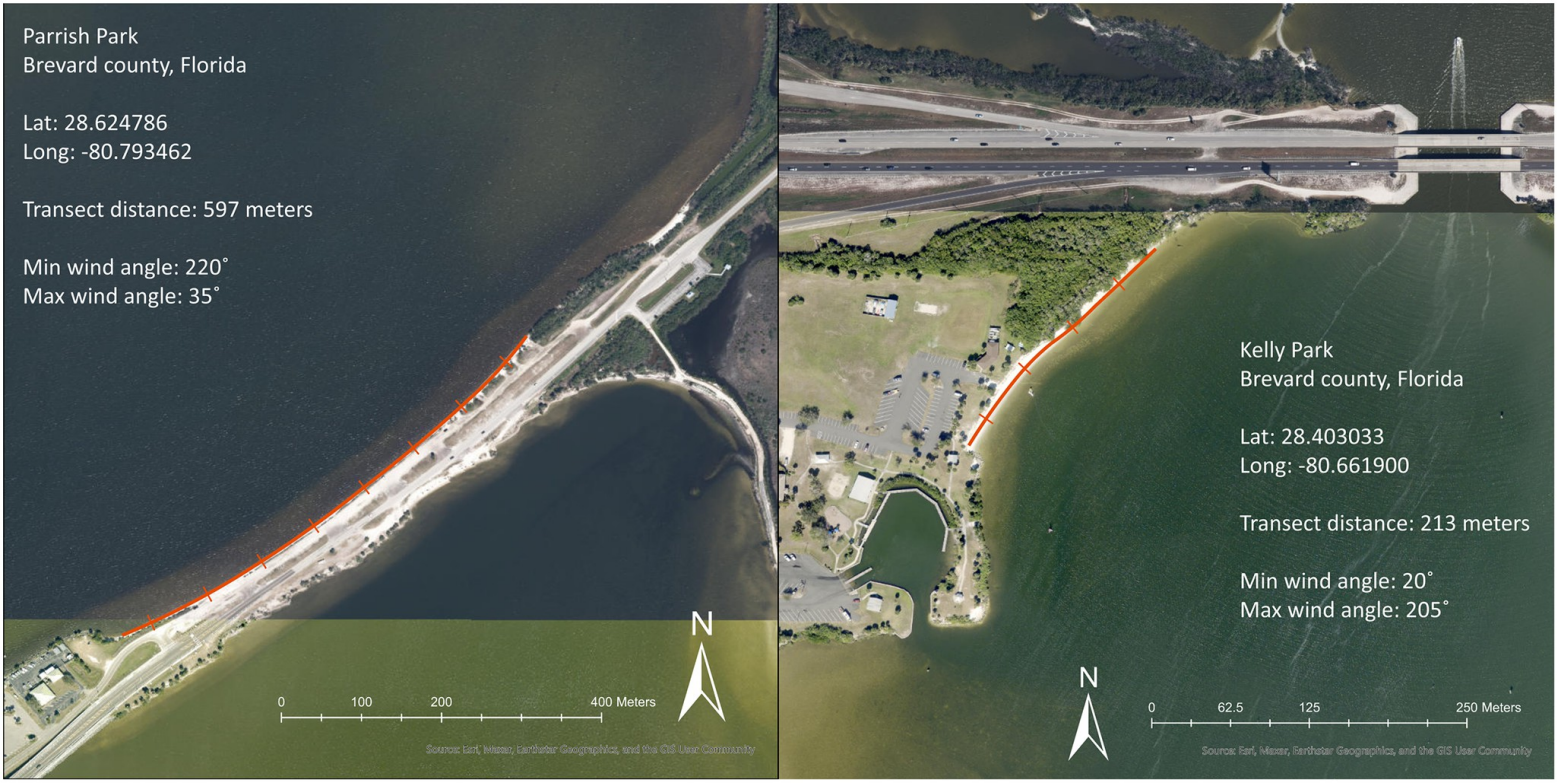
Orientation of survey sites and wind directions. Parrish Park and Kelly Park survey transects (indicated by red line) and site information, Brevard County, Florida. Created with ESRI ArcPro software 3.1.2. Content is the intellectual property of Esri and is used herein with permission. Copyright © 2024 ESRI and its licensors. All rights reserved.

Volunteers conducted surveys at two public shorelines in Brevard County, Florida, Kelly Park and Parrish Park. The study was performed with permission by Florida Fish and Wildlife Conservation Commission under the special activity license (SAL-17-1869-R). Kelly Park is a 6.2-ha park adjacent to the southwestern section of the Martin Andersen Beachline Expressway along the Banana River. The survey transect (213 m) was parallel to the shoreline (28.403033, -80.6619), (Figs 1 and 2). Parrish Park is a 14.8-ha park adjacent to the northeastern side of the Max Brewer Causeway. The survey transect (597 m) was parallel to the shoreline (28.62479, −80.7935). (Figs 1 and 2). Both Parrish Park and Kelly Park are located in the northern Indian River Lagoon which includes the widest sections of the lagoon, featuring the Banana River and Indian River water expanses (Fig 1).

### Data collection

**Fig 3:**
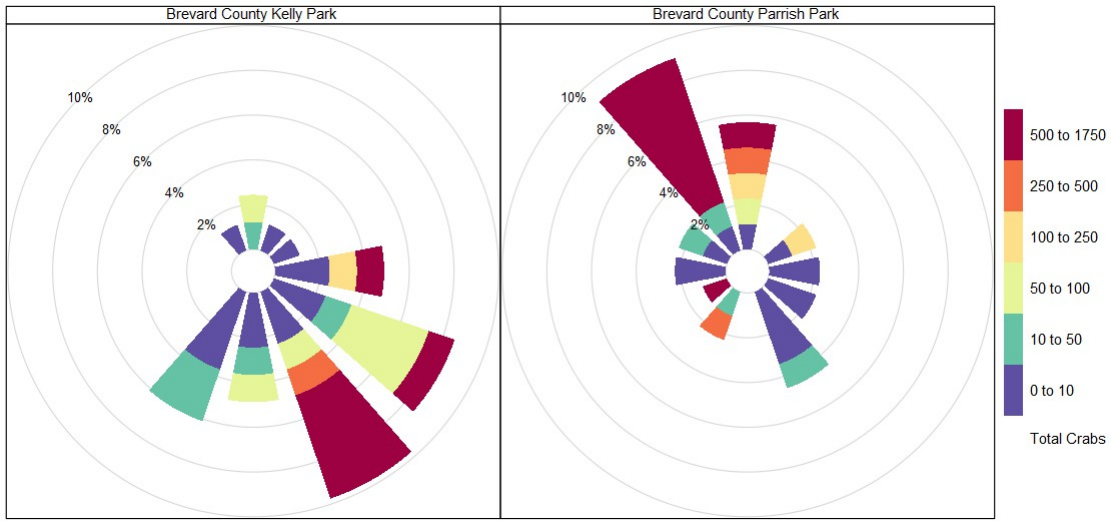
Wind rose graph of Parrish and Kelly Park survey sites. Wind rose graph depicting frequency of horseshoe crab count data at Kelly Park (top) Parrish Park (bottom) by wind direction and wind speed measured by anemometer.

Volunteers with Florida Horseshoe Crab Watch conducted daily surveys at the two sites beginning February 2^nd^, 2019, and ending April 30^th^, 2019, following methods as outlined by Heres et al. [15]. The timing of surveys followed yearly trends observed by Ehlinger et al. [12]. Volunteers surveyed at the time of the highest daytime tide at both locations. We determined the highest tide using National Oceanic and Atmospheric Administration’s (NOAA) tide-prediction service (https://tidesandcurrents.noaa.gov) [16]. Volunteers recorded the following information along the survey transects: total number of crabs observed, date, wind speed (m/s) and direction (°) using an anemometer, tide height (m) measured using USGS gauge data or NOAA tide prediction, salinity (ppm) using a refractometer, air and water temperature (°C) using a thermometer, and moon fullness (%) using the lunar calendar. We then assigned wind as either onshore or offshore in a direction based on the alignment of the shoreline, with an onshore wind blowing into the shore and an offshore wind blowing away from the shore. We observed wind direction trends visible in the raw data to confirm this method; for example, Parrish Park is aligned along the cross section of 220° and 35° degrees, so all wind that falls between that range is considered onshore, (Figs 2 and 3).

If the weather-monitoring devices at a site such as an anemometer or thermometer malfunctioned, we used data from the nearest weather station. We used the water height measured by the U.S. Geological Survey’s water gauge at Haulover Canal, near Mims, FL (gauge number 02248380; https://waterdata.usgs.gov/monitoring-location/02248380/; n = 162). When water height data could not be obtained, we used NOAA’s predicted tide height, which is determined using lunar and geological trends (n = 8) when a survey had missing data the survey was excluded from analysis (n = 3), hydrological modeling has shown that water height in the lagoon is primarily wind driven but correlated to oceanic trends [16, 17].

### Data analysis

We used a generalized linear regression model to assess the influence of environmental variables on horseshoe crab spawning at two Indian River Lagoon locations. The modeled response variable was the total number of spawning horseshoe crabs observed during each survey. Continuous predictor variables in the models were wind speed, tide height, moon %, water temperature, and air temperature. Categorical predictor variables were wind direction (onshore or offshore) and location (Parrish Park or Kelly Park). The global (all predictors included) model contained these continuous and categorical predictor variables in addition to a wind × direction interaction term. To account for the different shoreline lengths of the two survey locations (i.e., 597 m at Parrish Park and 213 m at Kelly Park), we included in each model an offset, log (Transect Length); hence, model parameter estimates and predictions are expressed as the mean number of horseshoe crabs per meter of shoreline at each location. Preliminary modeling based on the global model indicated evidence of overdispersion in the horseshoe crab count data; hence, we used negative-binomial regression instead of a more conventional Poisson regression. Additionally, we fit the global model with no interaction term to check for evidence of collinearity, and we excluded all variables with a variance inflation factor (VIF) >5 [18].

Nine negative-binomial regression models were evaluated, each representing a unique combination of continuous and categorical predictor variables Table 1. We assessed the relative support for each model using Akaike’s information criterion (AIC) [19] with a small-sample bias adjustment (AIC_c_) [20]. To facilitate model comparisons, we calculated Akaike weights, which range from 0 to 1, where the best-approximating model had the highest weight [21]. We identified the best-approximating set of models as those with summed Akaike weights totaling at least 0.95 (i.e., a 95% confidence model set) [21], and we considered parameter estimates to be important if their 95% confidence intervals did not overlap zero. To account for model selection uncertainty [21], we used the confidence model set to calculate model-averaged predictions of mean number of horseshoe-crabs per 100-m of shoreline at each location, along with unconditional standard errors and 95% confidence intervals. We based all inferences on parameter estimates and model-averaged predictions from the best-approximating set of models. Finally, we assessed goodness of fit for all models by evaluating model residuals using a simulation-based residual analysis implemented in the R package ‘DHARMa’ [22]. We conducted all analyses in R v.4.2.1 [23] using the packages ‘tidyverse’ (data processing and plotting) [24], ‘lubridate’ (data processing) [25], ‘glmmTMB’ (model-fitting) [26], and ‘MuMIn’ (model selection and model-averaged predictions) [27].

**Table 1:** Model names and predictor variables.

Model name	Predictor variables
Global Interaction	Wind + Direction + Tide + Moon + Water Temperature + Air Temperature + Location + Wind × Direction
Global	Wind + Direction + Tide + Moon + Water Temperature + Air Temperature + Location
No Wind	Tide + Moon + Water Temperature + Air Temperature + Location
No Tide Interaction	Wind + Direction + Moon + Water Temperature + Air Temperature + Location + Wind × Direction
No Tide	Wind + Direction + Moon + Water Temperature + Air Temperature + Location
No Wind or Tide	Moon + Water Temperature + Air Temperature + Location
Tide	Tide + Location
Wind Interaction	Wind + Direction + Wind × Direction + Location
Wind	Wind + Direction + Location

Model names and predictor variables included in the negative binomial regression models relating location and environmental predictors to the number of spawning horseshoe crabs at two Indian River Lagoon survey locations. All models included transect length as an offset to account for differences in survey transect length between locations.

## Results

**Fig 4:**
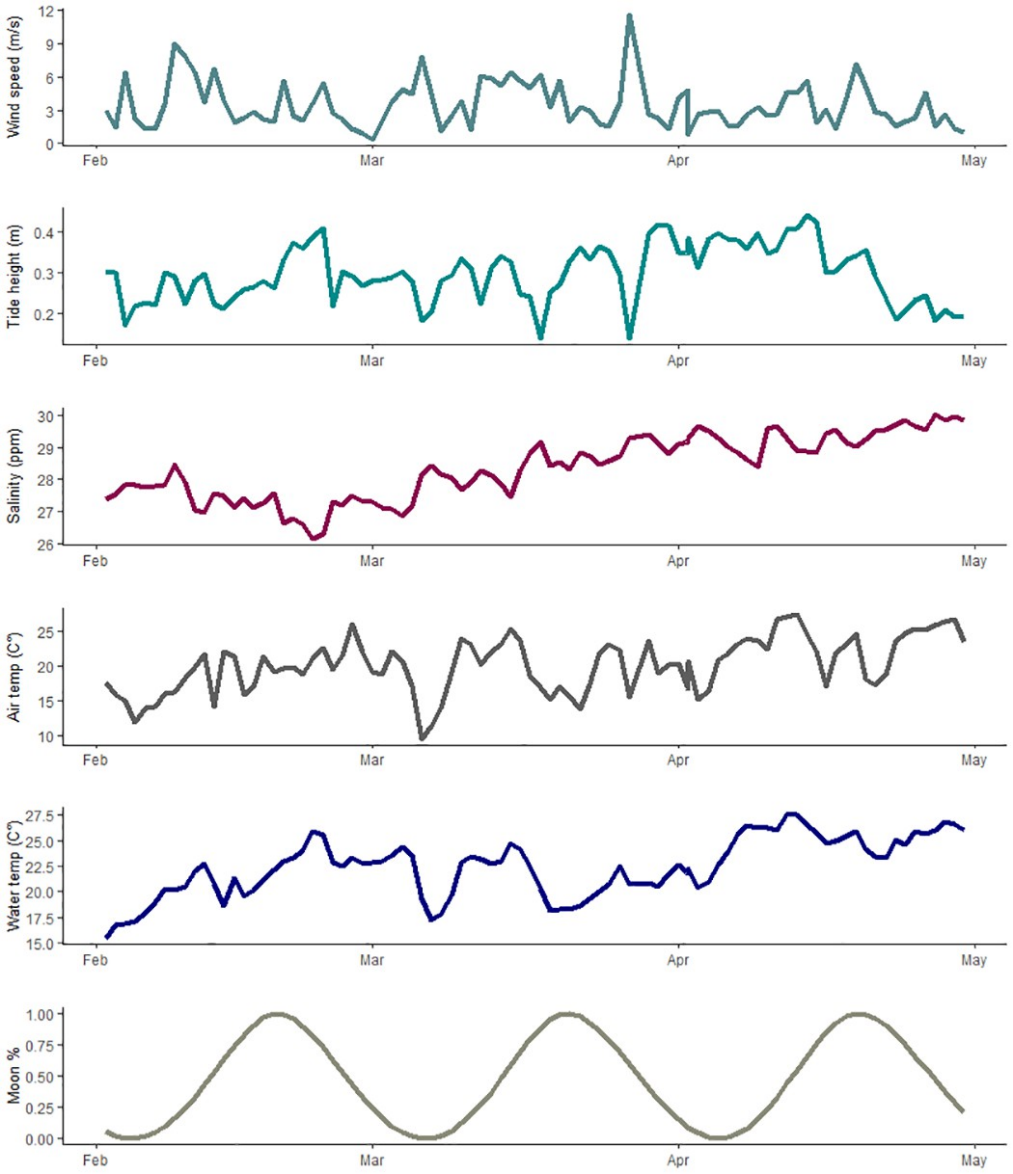
Environmental variables collected at Parrish Park. Environmental variables collected from Parrish Park, Brevard County, Florida from February 2019 –April 2019.

**Fig 5:**
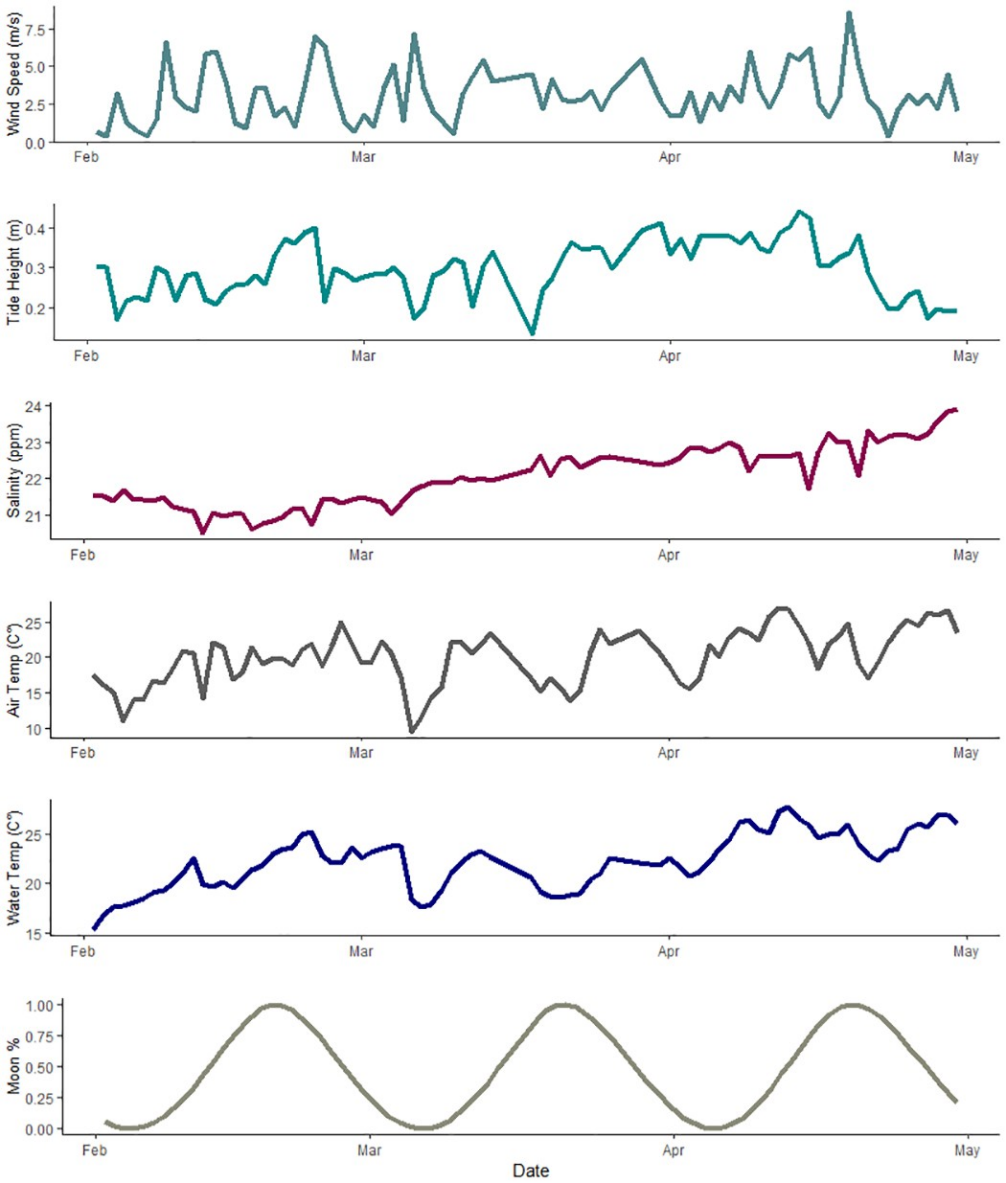
Environmental variables collected at Kelly Park. Environmental variables collected from Kelly Park, Brevard County, Florida from February 2019 –April 2019.

**Fig 6:**
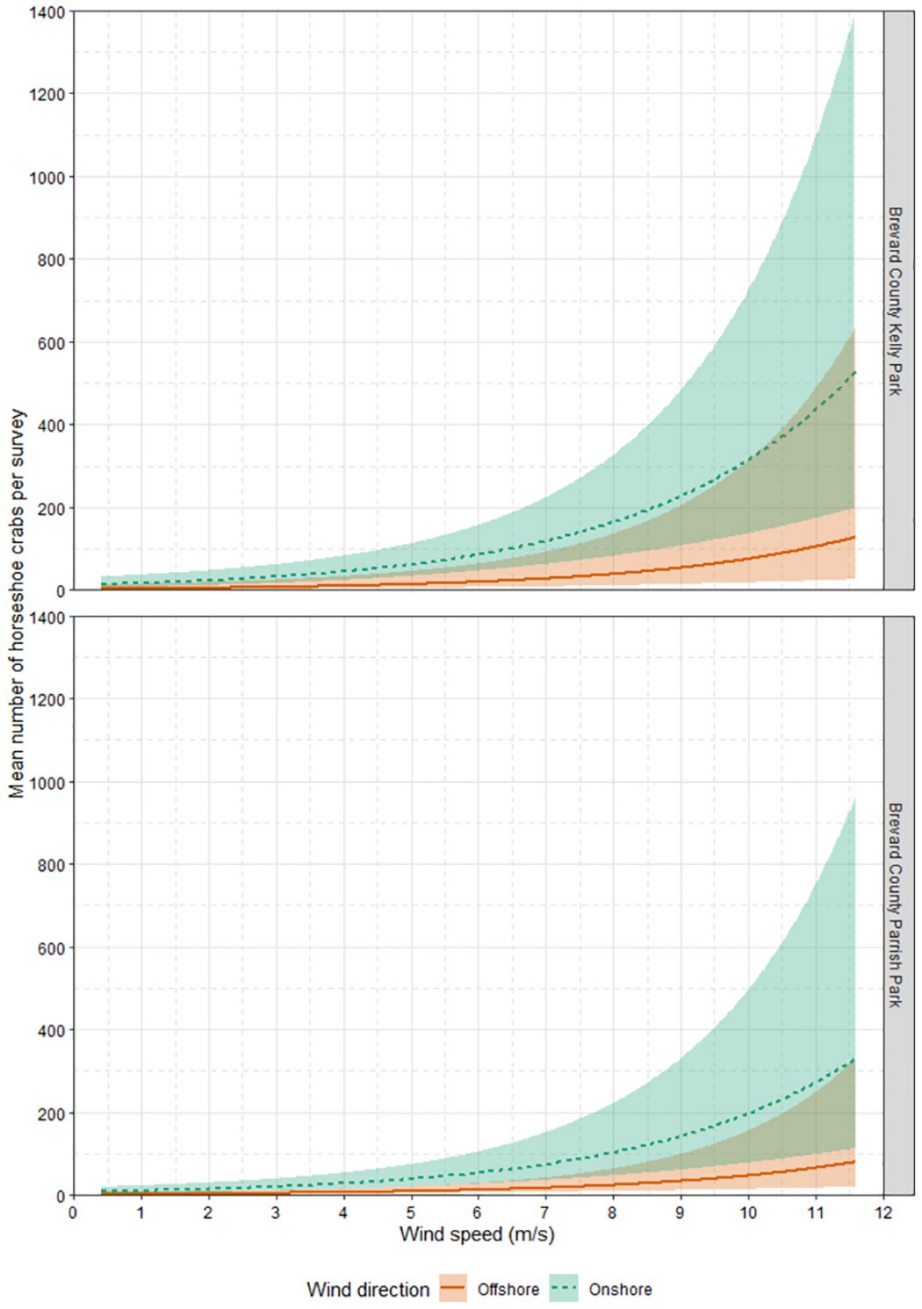
Model-averaged predictions of the mean number of horseshoe crabs at both survey sites. Model-averaged predictions of the mean number of horseshoe crabs (dotted and solid lines) and 95% confidence interval (shaded area) in relation to wind speed and direction at both study sites in the Indian River Lagoon, Florida. Predictions are based on the best-approximating set of negative binomial regression models and assume 100-m long surveys at Kelly Park and Parrish Park.

Florida Horseshoe Crab Watch volunteers conducted 170 surveys across both sites (Parrish Park, n = 88; Kelly Park, n = 82). We observed 10,518 crabs at Parrish Park and 6,476 crabs at Kelly Park. The number of horseshoe crabs present during each sampling event at Parrish Park (range: 0–1750; mean: 119.5; SD ±349.5) and Kelly Park (range: 0–1492; mean: 80.0; SD ± 251.9) was highly variable. The greatest numbers were observed at Parrish Park on February 13 (1,750), February 9 (1,464), and March 12 (1,323) and at Kelly Park on February 10 (1,492), February 20 (1,121), and February 21 (919), with smaller peaks at both locations throughout March and April (Figs 4 and 5). Wind speed and tide height were highly variable, but no clear pattern was discerned (Figs 4 and 5). Salinity, air temperature, and water temperature varied but generally increased throughout the study period (Figs 4 and 5). The number of observed horseshoe crabs was substantially greater when winds were onshore (northwest-Parrish Park, southeast-Kelly Park) (Fig 6).

### Negative-binomial-regression modeling

The simulation-based assessments of model residuals indicated that all candidate negative-binomial-regression models provided an adequate fit to the data, with no evidence of unexplained patterns (e.g., nonlinearities, VIF <5) in model residuals.

The best-approximating negative-binomial-regression model, “No Tide,” with an Akaike weight of 0.405, was 1.61 (0.405/0.251), 2.96 (0.405/0.137), 4.47 (0.405/0.091), and 4.94 (0.405/0.082) times more plausible than the second through fifth best-approximating models, respectively, based on ratios of their Akaike weights (Table 2). These five models made up the set of best-approximating models with cumulative Akaike weights summing to 0.97 (Table 2). Tide height, salinity, air temperature, water temperature, and percent moon fullness did not have a strong influence on mean numbers of spawning horseshoe crabs.

**Table 2:** Models names and outputs for candidate negative binomial regression models.

Model name	Predictor variables	K	AIC_c_	ΔAIC_c_	weight
No Tide	Wind + Direction + Moon + Water Temperature + Air Temperature + Location	8	910.032	0.000	0.405
Global	Wind + Direction + Tide + Moon + Water Temperature + Air Temperature + Location	9	910.989	0.956	0.251
No Tide Interaction	Wind + Direction + Moon + Air Temperature + Air Temperature + Wind × Direction + Location	9	912.201	2.168	0.137
Wind	Wind + Direction + Location	5	913.028	2.996	0.091
Global Interaction	Wind + Direction + Tide + Moon + Water Temperature + Air Temperature + Wind × Direction + Location	10	913.228	3.196	0.082
Wind Interaction	Wind + Wind × Direction + Location	6	915.003	4.970	0.034
Tide	Tide + Location	4	937.173	27.141	0.000
No Wind or Tide	Moon + Water Temperature + Air Temperature + Location	6	939.970	29.937	0.000
No Wind	Tide + Moon + Water Temperature + Air Temperature + Location	7	940.991	30.959	0.000

Model names, predictor variables, number of parameters (K), AIC_c_, ΔAIC_c_, and Akaike weights for the candidate negative binomial regression models relating location and environmental predictors to the number of spawning horseshoe crabs at two Indian River Lagoon survey locations. Parameter counts also include a model intercept and dispersion parameter (not shown).

Parameter estimates from models in the confidence set indicated that wind speed was positively related to the number of spawning horseshoe crabs, and this effect was generally consistent across the best-approximating set of models (Table 3). Model-averaged predictions indicated that the mean number of spawning horseshoe crabs increased 1.38 times for every 1 m/s increase in wind speed (Fig 6). Parameter estimates suggested onshore wind direction had a strong positive relationship with the mean number of spawning horseshoe crabs, although the effect of wind direction varied among the best-approximating set of models (Table 3). Model-averaged predictions indicated that the mean number of spawning horseshoe crabs was approximately 4.21 times greater during onshore winds than during offshore winds (Fig 6). Interestingly, there was little evidence that the effect of wind speed differed between onshore and offshore wind conditions. Based on ratios of Akaike weights, models that did not include the wind × direction interaction term were, on average, 2.90 times (0.405/0.137 = 2.96; 0.251/0.082 = 3.06; 0.091/0.034 = 2.68) as plausible as their counterparts that did include the interaction; moreover, the parameter estimate associated with the wind × direction interaction term was considered unimportant as its 95% confidence interval overlapped zero in all models in which it was included (Table 3).

**Table 3:** Model name, parameter estimates, Standard Errors (SE), and upper and lower 95% confidence limits.

*Model Name* and Parameter	Estimate	SE	Lower	Upper
*No Tide*				
Intercept	-5.849	1.389	-8.571	-3.127
Wind	0.326	0.059	0.211	0.442
Direction (Onshore)	1.513	0.419	0.691	2.335
Moon	0.086	0.373	-0.646	0.818
Water Temperature	0.012	0.071	-0.128	0.151
Air Temperature	0.095	0.054	-0.010	0.200
Location (Parrish Park)	-0.444	0.317	-1.066	0.177
Dispersion	7.074			
*Global*				
Intercept	-6.061	1.392	-8.788	-3.333
Wind	0.333	0.061	0.212	0.453
Direction (Onshore)	1.560	0.423	0.731	2.388
Tide	1.978	1.776	-1.503	5.459
Moon	0.082	0.370	-0.643	0.807
Water Temperature	-0.005	0.072	-0.146	0.136
Air Temperature	0.092	0.054	-0.013	0.198
Location (Parrish Park)	-0.423	0.319	-1.047	0.202
Dispersion	7.084			
*No Tide Interaction*				
Intercept	-5.991	1.508	-8.947	-3.035
Wind	0.360	0.149	0.069	0.651
Direction (Onshore)	1.689	0.830	0.063	3.315
Moon	0.083	0.374	-0.650	0.815
Water Temperature	0.012	0.071	-0.127	0.152
Air Temperature	0.094	0.054	-0.011	0.200
Location (Parrish Park)	-0.446	0.317	-1.067	0.175
Wind × Direction	-0.040	0.161	-0.355	0.275
Dispersion	7.080			
*Wind*				
Intercept	-2.791	0.564	-3.897	-1.685
Wind	0.299	0.056	0.189	0.408
Direction (Onshore)	0.906	0.369	0.182	1.630
Location (Parrish Park)	-0.819	0.289	-1.385	-0.253
Dispersion	7.148			
*Global Interaction*				
Intercept	-6.139	1.505	-9.088	-3.190
Wind	0.352	0.152	0.054	0.650
Direction (Onshore)	1.660	0.841	0.011	3.309
Tide	1.958	1.785	-1.541	5.457
Moon	0.080	0.370	-0.646	0.806
Water Temperature	-0.005	0.072	-0.146	0.137
Air Temperature	0.092	0.054	-0.014	0.197
Location (Parrish Park)	-0.425	0.319	-1.050	0.200
Wind × Direction	-0.023	0.165	-0.347	0.301
Dispersion	7.088			

Model name, parameter estimates, standard errors (SE), and upper and lower 95% confidence limits for the confidence set of negative binomial regression models relating location and environmental predictors to the number of spawning horseshoe crabs at two Indian River Lagoon survey locations. All estimates are reported on the natural log scale.

The mean number of spawning horseshoe crabs was, on average, slightly greater at Kelly Park than at Parrish Park. The parameter estimate associated with location, although indicating a weak negative effect, was generally imprecise among the best-approximating models (i.e., its 95% confidence overlapped zero). The sole exception was the Wind model, which indicated that the mean number of spawning horseshoe crabs was approximately 2.27 times larger at Kelly Park compared to Parrish Park (Table 3); however, model-averaged predictions indicated that the mean number of spawning horseshoe crabs was only approximately 1.60 times larger at Kelly Park.

## Discussion

The present study examined the correlation between horseshoe crab spawning and environmental data. We demonstrated that increased wind speed and onshore wind direction were strongly associated with horseshoe crab spawning at two locations in the Indian River Lagoon (Fig 6). We did not observe a strong association between wind direction and wind speed as an interaction term (wind × direction), which may be due to our decision to consider wind as either onshore or offshore. We determined a wind to be onshore using the alignment of the shoreline exclusively, meaning that wind blowing at nearly a 90° angle to the shore would still be considered onshore. For future studies with only one site or sites with identical orientation, we suggest that wind direction be measured as a categorical variable using cardinal direction. Our study sites had different orientations, so a binary factor (onshore versus offshore) was the most sensible way to include wind direction as a predictor variable in our analysis. Nevertheless, we found a strong association of horseshoe crab spawning with both onshore wind direction and greater wind speed. These findings advance our understanding of and ability to predict horseshoe-crab spawning in microtidal areas, which for many years could be described only as aperiodic or determined by water depth independent of tidal rhythms [11–13].

There is ample evidence that higher tides, which in Florida tend to occur around the full and new moons during the spring and fall, generally provide the greatest level of water, which strongly improves nesting conditions for horseshoe crabs [3, 5, 28]. While other environmental factors were included in these analyses, there was no significant correlation with mean number of spawning horseshoe crabs and air nor water temperature. This contrasts with Cheng et al. 2016, who found that despite a lack of correlation with moon phase and high tide, spawning density increased with higher water temperatures. However, wind speed and direction were not included in Cheng et al. 2016 [2]. In the absence of substantial lunar tides, as seen in microtidal estuaries like the northern Indian River Lagoon, a similar environmental effect, of increased water level, is created by strong onshore winds, which pile water higher on the shore than at other times [13]. In nonmicrotidal areas of Florida, in addition to increased high tide height, onshore wind direction and increased wind speed are correlated with spawning [3]. Conversely, in Delaware Bay, a northern Atlantic nonmicrotidal bay, higher onshore winds were negatively associated with spawning [29]. These studies, in conjunction with our findings, indicate that tidal range may be an important factor in addition to wind speed and direction. For example, Delaware Bay has a tidal range of 1.65 m, while Seahorse Key, Florida, has a range of 1.15 m [3, 28]. It is possible that there is an upper limit of wind speed that may lead to reduced spawning, due to increased wave action, as was seen in Delaware Bay. However, we did not observe an upper limit of wind in our study.

We suspect that in areas not described as microtidal but with a small tidal range, wind and wind direction are an important predictor for spawning. Horseshoe crab studies closer to the equator consistently find year-round spawning—in the southernmost part of the American horseshoe crab’s range, on the Yucatan peninsula, for example [30]. Other horseshoe crab species with distributions near the equator, such as *Carcinoscorpius rotudicauda* and *Tachypleus gigas*, are also reported to spawn year-round in areas with much smaller, and potentially microtidal tidal ranges [31]. We used this research to modify Florida Horseshoe Crab Watch sampling procedures of spawning beaches in the IRL, to include wind predicted surveys. The new survey design relies on close weather monitoring and flexibility within the volunteer monitoring network to ensure surveys are conducted during wind events in spawning season. In these study locations, volunteers are emailed prior to a wind event and asked to sign up to survey based on weather and wind forecasts. Since this is the typical method of organizing volunteers in this location, they are “on call” to survey. We propose that researchers conducting population studies of spawning horseshoe crabs consider wind conditions in addition to tide height and time of year when scheduling surveys, especially in microtidal ranges.

## Supporting information

S1 Data (CSV)
